# Books and Magazines

**Published:** 1910-02

**Authors:** 


### Books and Magazines.

---

ARTHROSTEOPEDIC SURGERY. Surgery of Extremities and Skeleton. By Stewart S. McCurdy, A. M., M. D., Arthopedic Surgeon to Columbia and Presbyterian Hospitals; Professor of Anatomy and Oral Surgery, Pittsburg Dental College (Univ. Pittsburg). Price, $1.00. Medical Abstract Pub. Co., Pittsburg, Pa.

This pretty little book, 3x5½ inches, in flexible black kid and gilt, is an abstract of the text-books on arthrosteopedic surgery—surgery of the extremities and skeleton. It is one of a series now very popular for reference. It supersedes the reference hand-book and the note-book and can be carried in the vest pocket.

---

PHYSICIANS' YEAR BOOK, 1910. Being a daily memorandum together with a miscellaneous jumble of facts and suggestions of interest and assistance to physicians. Sent free with the compliments of the publishers—on request, naming the TEXAS MEDICAL JOURNAL. M. J. Breitenbach Co., 53 Warren Street, New York.

---

A PRACTICAL STUDY OF MALARIA. By William H. Deaderick, M. D., Member American Society of Tropical Medicine; Fellow London Society of Tropical Medicine and Hygiene. Octavo of 402 pages, illustrated. Philadelphia and London: W. B. Saunders Company, 1909. Cloth, $4.50 net; Half Morocco, $6.00 net.

This is a work that should prove of great interest to the profession of the South.

Written by a Southern physician, who is recognized as a first authority on the subject of malaria, the work will be found most practical throughout. Special stress is laid on diagnosis and treatment, and the whole subject is covered in a way that should prove of the greatest value to the general practitioner. The illustrations used are mostly original. Altogether we feel the book will be found a very satisfactory one.

---

BIOGRAPHIC CLINICS, VOL. VI. Essays Concerning the Influence of Visual Function, Pathologic and Physiologic, Upon the Health of Patients. By Geo. M. Gould, M. D., Former Editor *American Medicine;* Author of various medical dictionaries, "Borderland Studies," "Right-handedness," etc. Philadelphia: B. Blakiston's Son & Co. Price, $1.00.

T[illegible] is a rather remarkable book; out of the ordinary. Its object seems to be to emphasize the significance of various eye troubles as affecting the bodily and mental health of people, especially children, and the importance of correctly understanding the causes, the pathology, and proper and timely treatment. Dr. Gould thinks that what are usually denominated reflex affections of the eye are really the disease, and that the "reflex" is just the other way. In former days teething was accused of every kind of trouble a baby had. There seems to be a tendency here to attribute even digestive troubles to "eye strain."

---

PRIMER OF SANITATION. By John W. Ritchie, Professor of Biology, College of William and Mary, Virginia, illustrated. Cloth. Post price, 40 cents. The World Book Company, Yonkers-on-Hudson, New York.

The purpose of this book is to teach the fourth and fifth grade pupil what he himself can do to keep his body in health. It is a simple work on disease germs and how to fight them. The author writes in a simple manner, presenting his subject in a form suitable for school use, elucidating the more important facts in regard to germ diseases and their prevention. It is as interesting as it is important. The teaching of sanitation in all schools should be compulsory.

---

THE PHYSICIAN'S POCKET ACCOUNT BOOK. By J. J. Taylor, M. D. Bound in full leather; 24 pages of practical instructions for physicians; 216 pages of accounts. Price, $1.00 per copy. Published by The Medical Council, 4105 Walnut St., Philadelphia, Pa.

This book is without a doubt the most complete and at the same time simple and thoroughly efficient account book that has ever been devised. Furthermore, it is absolutely legal and can be presented in any court of justice. It does not make use of any hieroglyphics, but everything is entered in plain language, and any judge can understand it.

